# Optimizing Digital Tools for the Field of Substance Use and Substance Use Disorders: Backcasting Exercise

**DOI:** 10.2196/46678

**Published:** 2023-12-12

**Authors:** Florian Scheibein, Elsa Caballeria, Md Abu Taher, Sidharth Arya, Angus Bancroft, Lisa Dannatt, Charlotte De Kock, Nazish Idrees Chaudhary, Roberto Perez Gayo, Abhishek Ghosh, Lillian Gelberg, Cees Goos, Rebecca Gordon, Antoni Gual, Penelope Hill, Iga Jeziorska, Eliza Kurcevič, Aleksey Lakhov, Ishwor Maharjan, Silvia Matrai, Nirvana Morgan, Ilias Paraskevopoulos, Zrinka Puharić, Goodman Sibeko, Jan Stola, Marcela Tiburcio, Joseph Tay Wee Teck, Zaza Tsereteli, Hugo López-Pelayo

**Affiliations:** 1 School of Health Sciences South East Technological University Waterford Ireland; 2 Health and Addictions Research Group Institut d'Investigacions Biomèdiques August Pi i Sunyer University of Barcelona Barcelona Spain; 3 United Nations Office of Drugs and Crime Dhaka Bangladesh; 4 Institute of Mental Health Pandit Bhagwat Dayal Sharma University of Health Sciences Rohtak India; 5 School of Social and Political Science University of Edinburgh Edinburgh United Kingdom; 6 Department of Psychiatry and Mental Health University of Cape Town Cape Town South Africa; 7 Institute for Social Drug Research Ghent University Ghent Belgium; 8 International Grace Rehab Lahore School of Behavioral Sciences The University of Lahore Lahore Pakistan; 9 Correlation European Harm Reduction Network Amsterdam Netherlands; 10 Department of Psychiatry Postgraduate Institute of Medical Education and Research Chandigarh India; 11 Department of Family Medicine David Geffen School of Medicine University of California, Los Angeles Los Angeles, CA United States; 12 European Centre for Social Welfare Policy and Research Vienna Austria; 13 The National Centre for Clinical Research on Emerging Drugs Randwick Australia; 14 The National Drug and Alcohol Research Centre University of New South Wales Randwick Australia; 15 National Drug Research Institute Curtin University Melbourne Australia; 16 Department of Public Policy Institute of Social and Political Sciences Corvinus University of Budapest Budapest Hungary; 17 Eurasian Harm Reduction Association Vilnius Lithuania; 18 Humanitarian Action Charitable Fund St Petersburg Russian Federation; 19 Management Center Innsbruck Innsbruck Austria; 20 Network of Early Career Professionals in Addiction Medicine Seligenstadt Germany; 21 Kethea Ithaki Thessaloniki Greece; 22 Faculty of Dental Medicine and Health Osijek Bjelovar University of Applied Sciences Bjelovar Croatia; 23 Youth Organisations for Drug Action Warsaw Poland; 24 Head of the Department of Social Sciences in Health Directorate of Epidemiological and Psychosocial Research Mexico City Mexico; 25 DigitAS Project Population and Behavioural Science, School of Medicine University of St. Andrews St Andrews United Kingdom; 26 Alcohol and Substance Use Expert Group Northern Dimension Partnership in Public Health and Social Well-Being Tallinn Estonia

**Keywords:** substance use, substance use disorders, addictions, telemedicine, eHealth, digital tools, backcasting exercise, drug addiction, ethical frameworks, digital health

## Abstract

**Background:**

Substance use trends are complex; they often rapidly evolve and necessitate an intersectional approach in research, service, and policy making. Current and emerging digital tools related to substance use are promising but also create a range of challenges and opportunities.

**Objective:**

This paper reports on a backcasting exercise aimed at the development of a roadmap that identifies values, challenges, facilitators, and milestones to achieve optimal use of digital tools in the substance use field by 2030.

**Methods:**

A backcasting exercise method was adopted, wherein the core elements are identifying key values, challenges, facilitators, milestones, cornerstones and a current, desired, and future scenario. A structured approach was used by means of (1) an Open Science Framework page as a web-based collaborative working space and (2) key stakeholders’ collaborative engagement during the 2022 Lisbon Addiction Conference.

**Results:**

The identified key values were digital rights, evidence-based tools, user-friendliness, accessibility and availability, and person-centeredness. The key challenges identified were ethical funding, regulations, commercialization, best practice models, digital literacy, and access or reach. The key facilitators identified were scientific research, interoperable infrastructure and a culture of innovation, expertise, ethical funding, user-friendly designs, and digital rights and regulations. A range of milestones were identified. The overarching identified cornerstones consisted of creating ethical frameworks, increasing access to digital tools, and continuous trend analysis.

**Conclusions:**

The use of digital tools in the field of substance use is linked to a range of risks and opportunities that need to be managed. The current trajectories of the use of such tools are heavily influenced by large multinational for-profit companies with relatively little involvement of key stakeholders such as people who use drugs, service providers, and researchers. The current funding models are problematic and lack the necessary flexibility associated with best practice business approaches such as lean and agile principles to design and execute customer discovery methods. Accessibility and availability, digital rights, user-friendly design, and person-focused approaches should be at the forefront in the further development of digital tools. Global legislative and technical infrastructures by means of a global action plan and strategy are necessary and should include ethical frameworks, accessibility of digital tools for substance use, and continuous trend analysis as cornerstones.

## Introduction

The 21st century has been marked by rapid technological and societal changes brought by the increasing availability of the internet [1,2], mobile phone network coverage [3], social media [4], virtual reality [5], machine learning, and related artificial intelligence [6]. Technology-induced disruptive changes are emerging across sectors, including health care [7], employment [8], research or education [9], and government or public administration [10]. These changes have led to an increasing need to manage the ethical, health, and societal impacts of such technologies [1,11,12], their practical implementation [13], and future impacts [14,15]. In this context, this study backcasts a future where digital tools are used optimally in the field of substance use by 2030.

Approaches in the field of substance use include prevention, early intervention, harm reduction, treatment, and recovery. A wide range of emerging digital tools influence these approaches in the domains of research, service provision, and policy making, including apps [16], chatbots [17], algorithms [18], dashboards [19], new service modalities [20-23], and use of digital tools for substance use [24-27]. The digitalization of this field brings with it the promise of extended access to services and more efficient distribution of limited expertise by means of telemedicine [28], better public health intelligence concerning substance use trends through data linkage studies [29], predictive models of substance use–related risks through artificial intelligence algorithms [30], improved provision of harm reduction interventions [31,32], and more effective prevention and early interventions for hard-to-reach populations [33,34]. Nevertheless, there are significant concerns to be considered, particularly concerning data sharing or protection, content moderation, informed consent, and access.

There is an arguably strong legal basis for data protection in many jurisdictions (eg, the General Data Protection Regulations in Europe), but large multinational for-profit companies are often still de facto protagonists in Europe and elsewhere concerning data regulation and control. Conversely, public health data sharing is frequently poor due to motivational, economic, and other barriers [35], including in the field of substance use due to the sensitive nature of these data [36]. The risk of generative artificial intelligence–generated content, coupled with a reduction in the content moderation workforce, has generated worries for public health experts in terms of the spread of misinformation, disinformation, and fake news [37]. In parallel, concerns have been raised that recent moves to restrict application programming interface access to social media platforms will limit both access to relevant public health data and people in the case of public health emergencies [37].

Issues such as social media content moderation [21,38] and related legal restrictions at the national level affect web-based and offline service delivery. Moreover, if substance use service delivery will become more digitally dependent in the future, it is necessary to anticipate and reconsider the impact of socioeconomic disadvantage and its impact on service access at regional, national, and global levels [39]. The ongoing digitalization of the substance use sector has thus created a range of specific challenges [20-22].

There is a global drive to create a better world by 2030 through the achievement of the various sustainable development goals adopted by the United Nations Sustainable Development Summit held in September 2015. This study, by means of a backcasting exercise with key stakeholders, aims to identify the key values, challenges, and facilitators toward achieving a future where digital tools are used optimally in the field of substance use by 2030. It also seeks to identify the essential cornerstones for realizing this vision and, in the process, contributes to nudging key stakeholders to achieve this aim by providing the foundations for a roadmap for the future.

## Methods

### Study Design

A backcasting exercise methodology was chosen as a foresight method to address complex and persistent “wicked” problems [40], where change is deemed necessary. The substance use phenomenon can be considered as a wicked problem due to the heavily politicized nature of this field [41], the consequent challenges to the legitimacy of the complex health care issues faced by this group [42,43], and the rapidly changing substance use trends.

Addressing the issues associated with this phenomenon requires approaches that take into consideration a diverse range of stakeholders with different (sometimes opposing) values, institutional complexity, and gaps in the existing knowledge [44]. In this context, our backcasting exercise involved key stakeholders, including people who use drugs, researchers, clinicians, and policy makers to cover the topics of ethics, human rights, effectiveness, sustainability, and effective long-term guidelines.

This method of backcasting used to explore the development of a standard joint unit by the coauthors of this paper was developed by López-Pelayo et al [45]. The coauthors drafted a current scenario that outlined the lack of consensus around a standard joint unit, a future scenario, and the key values, challenges, and facilitators, which should be considered. During a workshop at Lisbon Addictions 2022 conference, the current scenario, future scenario, values, challenges, and facilitators as well as the key milestones and cornerstones on the projected journey toward consensus were defined. Our backcasting exercise introduced 2 additional steps: (1) the current scenario and future scenario were drafted by a group of experts in the working group 1 on global issues as part of the Inter·GLAM (Global Perspectives on Addictions and Drug Market) project and (2) a web-based free open-source tool, Open Science Framework (OSF), was used both in advance and after the in-person workshop for web-based collaboration and refinement of terms and definitions. OSF has been promoted in addiction research [46], used for the storage of supplementary material [47,48], and to conduct addiction research [49]. In this project, OSF was used to overcome the limited in-person meeting time available for the conduct of this exercise—a limitation stressed by López-Pelayo et al [45].

### Sample Population and Study Settings

We recruited a convenience sample of professionals in academia, service delivery, and advocacy or policy making via the Inter·GLAM project. A core group developed the methodology of the exercise as well as the key components such as the draft versions of the values, barriers, facilitators, milestones, and cornerstones. The exercise began in May 2022 and ended on July 14, 2023. Lisbon Addictions 2022 was selected as the site for the backcasting exercise workshop, as it is one of the world’s largest subject matter conferences and was an official partner of the Inter·GLAM project. Subject matter experts in the fields of substance use service delivery, advocacy, academia, and policy were invited to participate. Those who participated in the exercise were people who primarily identified as clinicians (n=8), researchers (n=8), nongovernmental organization representatives (n=7), statutory authorities (n=2), and a person who uses drugs (n=1). However, several participants belonged to 2 or more categories.

### Backcasting Exercise

The backcasting exercise involved the following 5 steps.

#### Step 1 (Online): OSF Preparation

A dedicated OSF page was set up [50], which made all the exercise components publicly available. This page included draft components such as the methodology; current scenario; an ideal desirable 2030 scenario unbounded by circumstances, limitations, barriers, values, challenges, and facilitators; and a series of mini scenarios to introduce the following 5 distinct key areas aligning with relevant areas identified by the Inter·GLAM group as priorities: (1) web-based advertising, marketing, and health promotion; (2) availability, implementation, and sustainability of digital services; (3) innovations in digital tools; (4) data privacy, data sharing, and digital rights; and (5) web-based outreach with hidden populations.

#### Step 2: Introduction to the Exercise

The first part of the in-person session was used to explain the objectives, methodology, and expected outcomes of the exercise in the plenary setting. A description of the current scenario and a future ideal desirable 2030 scenario were presented to the participants, and time was allocated for questions and amendments.

#### Step 3: Prioritizing Relevant Areas

Participants were allocated to one of the 5 small multidisciplinary working groups, each focusing on one of the 5 key areas (identified in Step 1). Participants were allocated to the groups according to their area of interest or expertise. Participants included people who use drugs, service providers, nongovernmental organization advocacy groups members, and policy makers, with 5-6 people forming each group with 1 group for each of identified mini scenario. Stakeholders were provided with 3 printed lists of items relevant to optimizing digital tools to address substance use in 3 domains: values, challenges, and facilitators (Table 1 for definitions). The lists included definitions of each concept. Participants could also propose new items or suggest revisions for the provided definitions, if deemed necessary. Each working group was instructed to choose by consensus the 5 most relevant concepts from each list for each domain and record them on a flipboard. These concepts were then reported and discussed in plenary settings. Subsequently, the lead facilitators consisting of the working group leads (FS and MAT) and Inter·GLAM project coordinators (EC and HL-P) developed consensus in a plenary discussion to identify 5 values, challenges, and facilitators but not hierarchically ordered in terms of importance. Discussions were held until all the voiced opinions by the participants were adequately addressed. The participants could then further refine the concept definitions on the OSF page.

**Table 1:** Definitions of the core elements.

Term	Definition
Values	Beliefs, attitudes, and principles that may guide decision-making processes while shaping the desired future
Challenges	Obstacles, barriers, or difficulties that may need to be overcome to achieve the desired future
Facilitators	Resources, capabilities, and conditions that support and enable progress toward the desired future state

#### Step 4: Backcast Trajectories

Each group focused on a specific key area of the bigger desirable future scenario (see Step 1) by means of a mini scenario associated with their specific key area. The participants were asked to deconstruct the route starting from the end point in 2030 and moving backwards toward the present by using a predesigned canvas to facilitate the exercise. At the end of the exercise, the results were briefly discussed with the other members of the workshop.

#### Step 5: Defining Cornerstones and Milestones

Based on reflections during the exercise and the professional and personal background of the participants, a discussion was held within each working group regarding the milestones necessary for reaching the 2030 goals. Key terms and definitions were collated on OSF by using open Google documents to enable further definitions. These documents were circulated among those interested in further revision and discussion through the use of comments leading to a series of iterative revisions and definitions.

## Results

This section summarizes the results of the backcasting exercise conducted during the Lisbon Addictions 2022 conference and the subsequent iterative revisions on the OSF page. Participants identified 5 important values, challenges, and facilitators for achieving the 2030 goals in the optimal implementation of digital tools to address substance use (interventions) as well as a number of milestones for achieving them.

### Values Regarding Digital Tools for Substance Use

#### Summary

Five key values regarding digital tools to address substance use were identified by the group: (1) digital rights, (2) evidence-based tools, (3) user-friendliness, (4) access or availability, and (5) person-centeredness. More detailed definitions can be found in Table 2.

**Table 2:** Detailed definitions of the values regarding digital tools to address substance use.

Value	Definition
Digital rights	People should have freedom of expression, a right to privacy, a right to be free from harassment, and ownership of their data
Evidence-based tools	All digital tools should be informed by a continually evolving evidence base
User-friendliness	All digital tools should meet the needs of the key user group
Access or availability	No person should be excluded from digital tools, for example, through issues around digital divide, language, digital competency, disability, or gender
Person-centered	All tools should be focused on the person who will use the technology or tool

#### Digital Rights

Participants stressed the importance of promoting and protecting digital rights in the context of ongoing debates around content moderation in social media. Harm reduction organizations, for instance, experience difficulties because of content blocking while delivering services and providing information online. Innovations related to artificial intelligence–led identification of people who may be susceptible to intervention offers to treatment were also mentioned, along with the potential use of digital technologies to identify people who use drugs by governments that are compliant with nonhuman rights. Participants developed a consensus that the main goal of promoting and protecting digital rights was that digital tools are used in a way that will benefit people in a way that is ethical, safe, and secure.

#### Evidence-Based Tools

Participants emphasized the importance of founding approaches in the field of substance use on available, reliable, and high-quality evidence. However, questions were raised on the use of the current scientific methods (eg, the gold standard randomized clinical trial), which may not be suitable to develop the evidence base for rapidly evolving digital innovations, and new paradigms such as the Sequential Multiple Assignment Randomized Trial [51] or other trial methods were suggested as potential alternative methods to be considered [52,53]. A discussion also emerged on how to address and manage the wide availability of misinformation and disinformation related to substance use online.

#### User-Friendliness

According to participants, the user-friendliness of digital tools can impact their uptake, long-term use, and effectiveness, and thus this factor should be considered. The key requirements identified included being able to cater to the diverse needs of heterogeneous groups of people who use drugs with varying needs, language, and cultural context requirements.

#### Accessibility and Availability

The workshop participants highlighted that digital divide (understood as unequal access to digital technology) and low digital literacy (understood as a person’s ability to collect and assess information and engage with digital tools) continue to be significant barriers for a major portion of the global population. According to the participants, people in some regions of the world (most notably, low- and middle-income countries) continue to lack access to the internet, mobile phones, tablets and laptops, and new technologies that require advanced equipment (eg, virtual reality devices), which will likely pose new barriers to delivering and accessing digital interventions. Moreover, disadvantaged subpopulations in high- and middle-income countries experience similar barriers.

#### Person-Centeredness

The high heterogeneity in the needs of people who use drugs and of individuals living with drug dependency was another issue recognized by the working group. These diverse needs can include those related to particular substances, polysubstance use, routes of administration, specific populations, or environments. They may involve the parallel treatment of other (mental) health issues or chronic conditions, require addressing social determinants of health and economic disadvantages (eg, homelessness, poverty), and require responsiveness to local situations (eg, treatment availability, customs, norms, laws). In this context, experts argued that a person-centered approach should be adopted to the greatest possible extent.

### Challenges Regarding the Adoption of Digital Tools

#### Summary

Five key challenges regarding the adoption of digital tools for addressing substance use were identified: (1) ethical funding, (2) regulations, (3) commercialization, (4) best practice models, and (5) digital literacy and access or reach. More detailed definitions can be found in Table 3.

**Table 3:** Detailed definitions of the challenges in adopting digital tools for substance use.

Challenges	Definition
Ethical funding	Due to the bureaucratic and often lengthy procedures, funding coming from local and central governmental authorities and funders may be too slow to fund digital tools that are adequate and timely (in the context of changing drug markets and drug use patterns), especially where tools are global in nature. Digital decay is a big challenge, whereby technological solutions are not sustainably funded and decay over time.
Digital regulations	A range of digital rights issues such as the restriction of freedom of expression and rights to privacy or confidentiality and freedom from harassment have not yet been fully considered in this context. Certain laws and regulations may explicitly prohibit specific content (especially those related to harm reduction). For example, providing advice and information on harm reduction measures or safer dosing may be considered illegal in some jurisdictions. Care must also be taken to prevent the misuse of digital regulations or security laws to collect personal data at national and global levels.
Commercialization	Commercial interests are likely to advertise and market substances online to people who use drugs.
Lack of best practice models	A lack of best practice models results in a situation where digital service developers and service providers do not have clear points of reference that can be applied globally. This likely negatively impacts the effectiveness and quality of services operating. There is an inconsistent application of data protection restrictions, for example, strict rules related to data protection or privacy are not applied equally globally, and data sharing across regions and sectors is also highly variable. At a global level, many regional approaches (eg, Western, Russian, Chinese) are leading to the development of systems that lack compatibility or interoperability.
Digital literacy and access or reach	Target groups members may lack the digital literacy skills to use digital tools effectively. This problem may be especially profound among certain groups (eg, older adults).

#### Ethical Funding

The sustainable funding of programs and projects and the development of digital tools were identified as a key challenge across working groups, with precarity and instability of funding seen as a critical factor affecting long-term sustainability. The necessity of identifying sustainable sources of funding was discussed, while the importance of this funding being ethical was stressed, particularly in the context of possible industry involvement. Significant skepticism was expressed concerning the motives of for-profit entities, but it was also argued that there can be shared value or mutual interests around projects where public health and profit outcomes coalesce around shared objectives. Participants also noted that the current grant application processes for most funders do not adequately allow for business practices like market research (customer discovery, etc) and rather, the focus is on defining the population and features before the project begins. This means, unlike start-ups, it is difficult to pivot during a project to better address the need through a change in features or to switch to a different population that has the need for the features originally specified. Public funders may also be hesitant or resistant to fund certain types of technologies that are politically contentious. Participants argued that public entities are reluctant to fund the development of web-based harm reduction initiatives, as they are frequently seen as controversial.

#### Digital Regulations

Session participants highlighted several cases where harm reduction content was removed from social media platforms, for example, a recent case between SIN (Students Drug Policy Initiative) Poland and Facebook [38]. Concerns were also raised about predictive algorithms currently being developed to identify people with substance use disorders as treatment ready [54]. Experts encouraged more extensive work with stakeholders (such as social media companies) in the field of content moderation as well as advocacy efforts against current policies and laws, which may restrict the access to evidence-based information or advice.

#### Commercialization

Being cognizant of alcohol and gambling, participants expressed concerns that commercial interest companies are likely to advertise and market substances online once they become legal and may focus their marketing to those with an increased risk of substance use–related problems. The likely emergence of new licit industries (eg, Big Cannabis, Big Psychedelics) and their potential involvement in aggressive marketing was deemed worth monitoring and proactively responding to. It was also discussed whether the development and commercialization of digital tools in this field should be done exclusively by health or governmental institutions or also by private companies. Ethical issues are likely to arise from the involvement of big companies with economic interests, but it was also recognized that such actors may also offer bigger funding opportunities.

#### Lack of Best Practice Models

The group discussed the lack of availability of best practices for both developing and using digital tools, although attention was brought to emerging practices in the field of harm reduction such as Eurasian Harm Reduction Association's recommendations for setting up web-based harm reduction services [22] and Peer-to-Peer Counsellor Manual for Online Counselling [23] and the guide “Recommendations Web—outreach for people who use drugs” developed by the United Nations Office on Drugs and Crime [21]. Digital tools are equally being implemented in the substance use prevention and early intervention field by, for instance, the BePrepared team in Germany for young refugees with hazardous substance use [33]. The lack of available best practice was seen to negatively impact the quality assurance of service and highlighted the need for increased quality management. In this context, issues around the well-being of health care workers working in the web-based space using digital tools and the need for new management protocols to work with such potentially remote workers were discussed [23].

#### Digital Literacy and Access or Reach

The multidimensional nature of digital inequalities and the digital divide were stressed, including the importance of focusing on dimensions for any given scenario and the development of an understanding of boundary settings and challenges. It was also highlighted that we must remain cognizant of the potential role of hybrid approaches and the use of digital environments as a medium or setting as well as a tool. Digital literacy was highlighted as a key challenge, which may particularly affect nondigital natives (eg, older people), disadvantaged populations, as well as health sector representatives. In this context, disparity in terms of technology development, investment, and accessibility between high income and low- and middle-income countries was emphasized. The likely impact identified by participants was primarily concerned with people’s access to services and their ability to critically analyze available information. In addition to low digital literacy, the digital divide was also identified as an obstacle for some people to engage in web-based services due to poor internet access, lack of computer devices, or due to the lack of access to modern newly emerging technologies (eg, virtual reality headsets).

### Facilitators of Adopting Digital Tools

#### Summary

Five key facilitators of adopting digital tools for addressing substance use were identified: (1) scientific research, interoperable infrastructure, and a culture of innovation; (2) expertise; (3) ethical funding; (4) user-friendly design; and (5) digital rights and regulations. More detailed definitions can be found in Table 4.

**Table 4:** Detailed definitions of the facilitators of adopting digital tools for addressing substance use.

Facilitators	Definition
Scientific theory, infrastructure, and a culture of innovation	Increased focus, discussion, and adoption of open science (making science more open), citizen science (involving the public in science), outbreak science (identifying and managing outbreaks), and implementation science (putting evidence into practice). New digital tools and infrastructure for scientific processes (eg, communities of practice, data sharing).
Expertise	Expert working groups should be involved and include all key stakeholders. Guidelines could help key stakeholders manage various domains (eg, content moderation) and their various challenges (eg, ethical issues, data protection). Emerging best practices should be continuously shared and enable stakeholders to continually improve their practice. Standards could enable the certification of quality of services, and data sharing standards could enable the sharing of data according to Findable, Accessible, Interoperable, and Reusable data standards.
Ethical funding	Nonprofit and governmental or authority funding could help fund digital tools in this area.
User-friendly design	Technology that meets people’s needs are likely to increase adoption, use, and efficacy.
Digital rights and regulations	A greater focus on digital rights will help promote the optimal use of digital tools in this field.

#### Scientific Research, Interoperable Infrastructure, and a Culture of Innovation

Participants highlighted that there was a need for further collaboration among researchers, people who use drugs, clinicians, and advocacy groups in the conduct of research, development of infrastructure, and the promotion of innovation in this field. It was acknowledged that multidisciplinary research needs to be conducted at all stages from planning to execution to monitoring and evaluation. A need to ensure that technologies and infrastructure were interoperable was also identified. The importance of multistakeholder involvement in innovations in this field was also stressed. It was suggested that a culture of innovation would include lean or agile start-up methods used in business.

#### Expertise

The working group highlighted the need to build capacity and expertise of developers and end users for digital tool development and use. This includes the development of expert advisory groups composed of all key stakeholders, including people who use drugs that would help monitor and oversee efforts such as the cocreation of best practices and guidelines around cybersecurity, data sharing, content moderation, and ethical use of artificial intelligence.

#### Ethical Funding

A long discussion took place on the need for sources of ethical funding and the potential role of industry in the development of digital tools. Some experts indicated significant skepticism toward the involvement of for-profit entities’ and advocated for no industry involvement. Others argued for the involvement of industry where there was a shared value (mutual interests) around well-being and health. A consensus was achieved that all funding procedures should always consider ethical questions explicitly.

#### User-Friendly Design

All experts emphasized the importance of user-friendly designs of digital tools for substance use to enhance uptake, engagement or adherence, efficacy, and efficiency. It was noted that the term “user-friendly” is rather generic, and its specific features will vary significantly depending on the characteristics of specific target groups (eg, different age groups). However, some general and universal features of user-friendliness mentioned by the experts included easiness of use, availability in local languages, the use of simple language and terms, lack of excessively lengthy text descriptions, and accessibility for people with reading difficulties or cognitive difficulties. In terms of content, it was also advised to avoid scientific jargon and use common expressions or colloquialisms instead of scientific language to enhance clarity and understandability of information.

#### Digital Rights and Regulations

For the optimal use of digital tools, working groups stressed the importance of digital rights ensured by the existence of appropriate regulations and laws that are rooted in equity and human rights principles. It was considered extremely important to protect people who use drugs, service providers, and other key actors’ data privacy, confidentiality, right to transparent information, and health care provision while also protecting them from harassment from automated technologies engaging in predictive risk prediction and actions of state or nonstate malicious actors.

### Milestones or Cornerstones (2022-2030)

**Figure 1:**
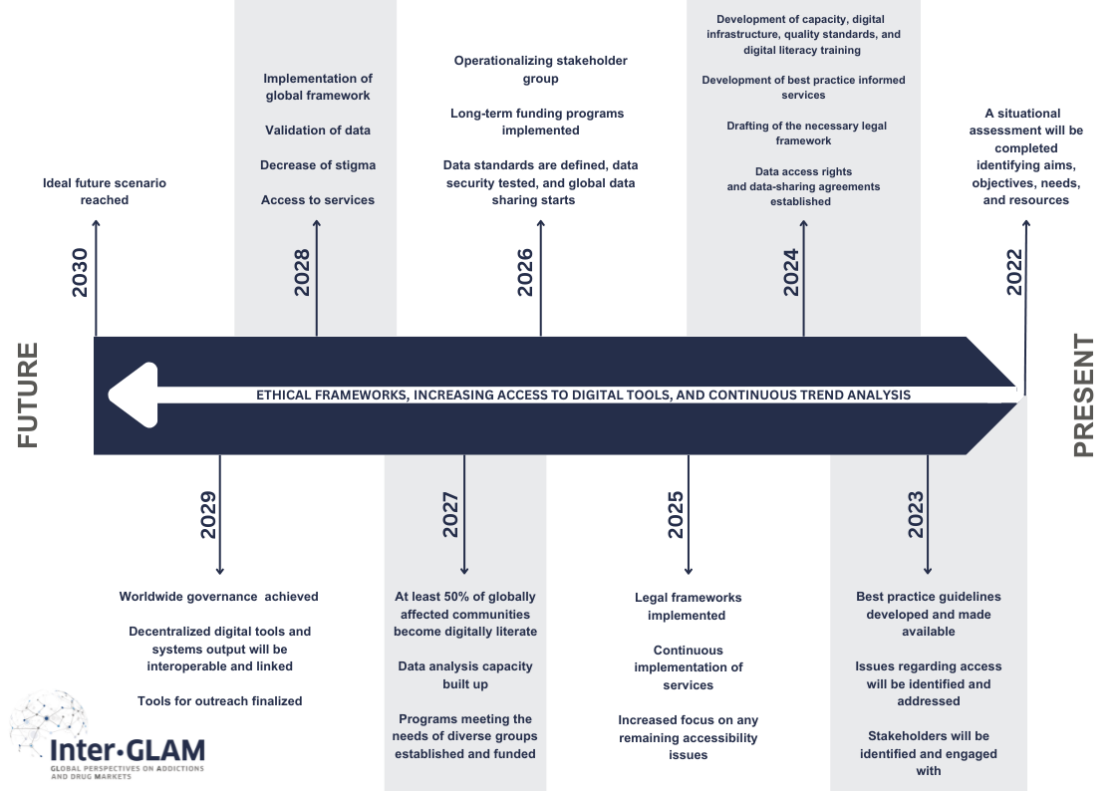
Milestones and cornerstones.

Several milestones to achieving the idealized future were proposed by participants as they moved from 2030 to the present. Three underlying cornerstones were also identified by participants (see Figure 1): ethical framework, increasing access to digital tools, and continuous trend analysis.

#### Ethical Framework

Participants proposed that an integrative and levelled ethical framework should inform substance use–related work in the digital space. This framework could guide social media operators on how to moderate content related to substance use, governments in creating appropriate regulations focused on the protection of individuals, as well as developers (either public or private entities) in the creation of digital tools.

#### Increasing Access to Digital Tools

Participants proposed that a range of focused and coordinated efforts should be undertaken to increase access to digital tools and reduce the digital divide. This includes efforts aiming to widen the geographical coverage of the internet network to improve internet access, enhance digital literacy among those less familiar with new technologies, and address accessibility issues related to physical and mental disabilities and different cognitive abilities, dependent on local and regional contexts.

#### Continuous Trend Analysis

Participants proposed that interventions, policies, and infrastructures should be subject to continuous monitoring and evaluation. Data sharing infrastructures could be regularly reviewed in terms of security and conformance with digital rights. Monitoring of digital tools would allow for assessment of their effectiveness and adequacy and for adjusting them accordingly to the dynamic changes in substance use. Services should be adaptive to changes in substance use patterns.

## Discussion

### Principal Results

In our study, the backcasting exercise to identify values, challenges, facilitators, and milestones or cornerstones for developing and implementing digital tools to address substance use turned out to be rich and informative. The participants in our study highlighted the importance of protecting people who use drugs and of service providers’ digital rights to privacy, confidentiality, security, freedom of expression, freedom of harassment, and high-quality person-centered health care. There is a strong need for developing a levelled ethical framework for a range of issues (open science, citizen science, and data sharing) [36], content moderation [38], and the use of algorithms, which predict the receptiveness of people living with substance use disorders to treatment [54]. Increasing access or availability and monitoring drug market trends continuously are also paramount cornerstones for the optimal use of digital tools in the field of substance use by 2030.

Access issues, ethical funding, user-friendliness, and digital rights were the recurring themes throughout the discussions. Concerted efforts may be needed to address issues associated with the digital divide for the effective use of digital tools [21]. The current public funding models may be problematic, as they often do not allow engagement with current best practices in technology development, such as the use of lean and agile approaches that are flexible in terms of the features and population served. Industry involvement continues to be a problem but may be circumvented by engaging in open science practices [55], which, however, remain poorly adopted in this field of substance use [36].

Well-established technologies such as mobile apps still often function suboptimally in this field. Many apps in this field currently lack an evidence base [56], frequently lack significant positive effects [56], and some may even encourage harmful use of substances [57]. Nevertheless, promising developments in the prevention and early interventions in this field have been identified [33]. Issues around content moderation also require more significant focus both in terms of removing harmful content [57-59] and preventing privately run content-moderation policies, thereby negatively impacting service provision [38].

Ensuring high-quality reliable data in this field is likely to also be impacted by newly emerging technologies such as large language models linked to generative artificial intelligence, for example, as best illustrated by the disruptive impact of ChatGPT, which is built using such models [60]. Generative artificial intelligence–based technologies will likely require human supervision in the near future to ensure the reliability and validity of information and the prevention of bias [61]. There may be significant risk for the spread of misinformation around substance use and substance use disorders and the replication of discrimination and stigmatization based on historical data used to train or teach or develop such large language models. In this context, there may be a need for greater involvement and capacity building of health care workers to counteract this type of misinformation to prevent the potential negative health impacts of the virulent spread of such information [62] and the replication of such biases.

Building the necessary ethical and technological infrastructure will require time and effort and multistakeholder engagement [63]. The investment in open science practices and the open sourcing of technology and data sets are likely to contribute substantially [36]. A “one-size-fits-all” for data sharing is unlikely to work, and multistakeholder data sharing occur through permissioned access systems, whereby different actors such as law enforcement officers and people who use drugs may be able to share and access different types of information and data, ranging from newly emerging trend data to the sharing of best practices [63].

### Limitations

There are several limitations to the backcasting exercise that we discuss in this paper. As mentioned in the methods section, the in-person exercise involved a purposive convenience sample of 26 professionals in the field of substance use who attended the Lisbon Addictions 2022 conference. Since they self-selected to take part in the exercise, many profiles were underrepresented, such as representatives of social media companies, health technology industry, prevention and early intervention field, all continents, and substance supply field. Nevertheless, considering that this was a pioneer and explorative exercise, the main aim of gathering a representative sample was not to be able to include all the emerging topics. We were also limited in the amount of time during the in-person workshop, wherein we had to rely on broad abstractions and definitions that are likely to have contributed to implicit assumptions around the definitions of key terms and concepts. However, the backcasting exercise yielded information that should be brought to a broader audience for discussion and refinement. Future research could, for instance, focus on the development in specified substance use fields and refine the defined key values, facilitators, challenges, and milestones accordingly.

### Conclusion

The use of digital tools in the field of substance use may be linked to a range of risks and opportunities that need to be managed. Trajectories of the use of such tools are currently heavily influenced by large multinational for-profit companies, with relatively little involvement of key stakeholders such as people who use drugs, service providers, and academicians. A Global Action Plan and Strategy could help minimize the risks and maximize the benefits associated with the use of digital tools in this space. Our backcasting exercise suggests that such an action plan and strategy should be based around key principles, including the promotion of access or availability, digital rights, user-friendly design, and person-focused approaches. Addressing the digital divide and ensuring ethical and sustainable funding are the key issues that will need to be considered in more detail. The adoption of successful business practices such as the use of reflexive lean and agile approaches as well as the use of customer discovery techniques such as engaging with key stakeholders (people who use drugs, service providers, nongovernmental organizations, authorities, and policy makers) would likely benefit this field. Expertise must be developed among all stakeholders based on a shared firm and levelled ethical framework. Continuous trend analysis of substance use should inform global approaches in digital tools development.

## Abbreviations

GLAM: Global Perspectives on Addictions and Drug Market
OSF: Open Science Framework
SIN: Students Drug Policy Initiative
